# Micro-RNA profiles of pathology and resilience in posterior cingulate cortex of cognitively intact elders

**DOI:** 10.1093/braincomms/fcae082

**Published:** 2024-03-07

**Authors:** Christy M Kelley, Bryan Maloney, John S Beck, Stephen D Ginsberg, Winnie Liang, Debomoy K Lahiri, Elliott J Mufson, Scott E Counts

**Affiliations:** Department of Translational Neuroscience and Neurology, Barrow Neurological Institute, St. Joseph’s Hospital and Medical Center, Phoenix, AZ 85013, USA; Departments of Psychiatry and Medical and Molecular Genetics, Indiana University School of Medicine, Indianapolis, IN 46202, USA; Departments of Translational Neuroscience and Family Medicine, Michigan State University College of Human Medicine, Grand Rapids, MI 49503, USA; Center for Dementia Research, Nathan Kline Institute, Orangeburg, NY 10962, USA; Departments of Psychiatry, Neuroscience & Physiology, and the NYU Neuroscience Institute, New York University Grossman School of Medicine, New York, NY 10016, USA; Translational Genomics Research Institute, Phoenix, AZ 85004, USA; Departments of Psychiatry and Medical and Molecular Genetics, Indiana University School of Medicine, Indianapolis, IN 46202, USA; Department of Translational Neuroscience and Neurology, Barrow Neurological Institute, St. Joseph’s Hospital and Medical Center, Phoenix, AZ 85013, USA; Departments of Translational Neuroscience and Family Medicine, Michigan State University College of Human Medicine, Grand Rapids, MI 49503, USA

**Keywords:** posterior cingulate cortex, micro-RNA, cognition, resilience, transcriptomics

## Abstract

The posterior cingulate cortex (PCC) is a key hub of the default mode network underlying autobiographical memory retrieval, which falters early in the progression of Alzheimer’s disease (AD). We recently performed RNA sequencing of post-mortem PCC tissue samples from 26 elderly Rush Religious Orders Study participants who came to autopsy with an ante-mortem diagnosis of no cognitive impairment but who collectively displayed a range of Braak I–IV neurofibrillary tangle stages. Notably, cognitively unimpaired subjects displaying high Braak stages may represent cognitive resilience to AD pathology. Transcriptomic data revealed elevated synaptic and ATP-related gene expression in Braak Stages III/IV compared with Stages I/II, suggesting these pathways may be related to PCC resilience. We also mined expression profiles for small non-coding micro-RNAs (miRNAs), which regulate mRNA stability and may represent an underexplored potential mechanism of resilience through the fine-tuning of gene expression within complex cellular networks. Twelve miRNAs were identified as differentially expressed between Braak Stages I/II and III/IV. However, the extent to which the levels of all identified miRNAs were associated with subject demographics, neuropsychological test performance and/or neuropathological diagnostic criteria within this cohort was not explored. Here, we report that a total of 667 miRNAs are significantly associated (rho > 0.38, *P* < 0.05) with subject variables. There were significant positive correlations between miRNA expression levels and age, perceptual orientation and perceptual speed. By contrast, higher miRNA levels correlated negatively with semantic and episodic memory. Higher expression of 15 miRNAs associated with lower Braak Stages I–II and 47 miRNAs were associated with higher Braak Stages III–IV, suggesting additional mechanistic influences of PCC miRNA expression with resilience. Pathway analysis showed enrichment for miRNAs operating in pathways related to lysine degradation and fatty acid synthesis and metabolism. Finally, we demonstrated that the 12 resilience-related miRNAs differentially expressed in Braak Stages I/II versus Braak Stages III/IV were predicted to regulate mRNAs related to amyloid processing, tau and inflammation. In summary, we demonstrate a dynamic state wherein differential PCC miRNA levels are associated with cognitive performance and post-mortem neuropathological AD diagnostic criteria in cognitively intact elders. We posit these relationships may inform miRNA transcriptional alterations within the PCC relevant to potential early protective (resilience) or pathogenic (pre-clinical or prodromal) responses to disease pathogenesis and thus may be therapeutic targets.

## Teaser

Kelley *et al*. quantified micro-RNAs in the posterior cingulate cortex from cognitively intact elders with Braak Stages I–IV; higher Braak stages represented cognitive resilience. A total of 667 micro-RNAs correlated with ante-mortem cognitive test scores and/or neuropathological diagnosis. Twelve micro-RNAs differentiated Braak Stages I/II versus III/IV and were predicted to target amyloid, tau and inflammation pathways.

## Graphical Abstract

**Graphical Abstract:**
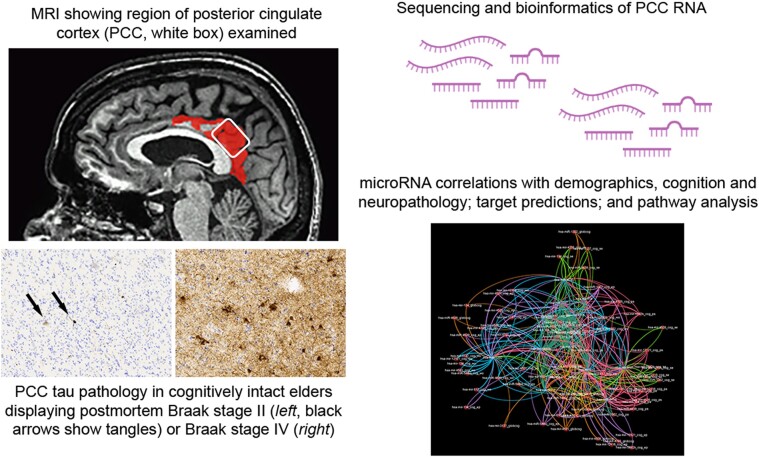

See Soreq (https://doi.org/10.1093/braincomms/fcae099) for a scientific commentary on this article.

## Introduction

Alzheimer’s disease (AD) is the most common cause of dementia in the elderly, manifesting as memory, language and attentional/executive function impairments and, progressively, total loss of independence and quality of life.^1-3^ Neuropathologically, AD is characterized by the presence of extracellular plaques containing aggregates of amyloid-β (Aβ) peptides, intracellular neurofibrillary tangles (NFTs) containing aggregates of hyperphosphorylated, misfolded moieties of the protein tau,^4,5^ neuroinflammation, neuronal loss and synaptic dysfunction.^6-8^ The extent of neuron loss and synaptic defects distinguishes people with mild cognitive impairment (MCI) or AD from those with intact cognitive abilities.^9-12^ By contrast, the extent of amyloid plaque and NFT pathology among these groups is equivocal for predicting cognitive status. For instance, older individuals with an ante-mortem clinical diagnosis of no cognitive impairment (NCI) display Braak NFT stages ranging from I to VI upon post-mortem neuropathological evaluation,^13,14^ which places these cases within the spectrum of the neuropathological classification schema of AD. This suggests considerable neuropathologic heterogeneity in elderly individuals with NCI and that those with high NFT burden may be resilient in the face of AD pathology.^15,16^ Therefore, identifying the molecular mechanisms differentiating NCI subjects who display low versus high NFT loads may provide tangible clues underlying cognitive resilience and inform intervention strategies to delay disease onset and/or slow disease progression during a clinically efficacious timeframe.^17^

The activity of small non-coding micro-RNAs (miRNAs) that regulate mRNA stability^18^ is an underexplored potential mechanism of resilience through the fine-tuning of gene expression within complex cellular networks.^19,20^ Select miRNAs regulate diverse brain functions including neurogenesis and differentiation, neurophysiology and synaptic plasticity, and energy metabolism.^21-24^ This widespread influence of miRNA regulation on neuronal physiology suggests that perturbations in miRNA function could be involved in the complex molecular pathogenesis of neurodegenerative disorders including MCI and AD.^19,20^ Indeed, MCI and AD brains display altered expression of several miRNAs that regulate Aβ metabolism, innate immunity, neurophysiology and synaptic function, and neuronal survival.^25-34^ However, whether miRNA networks are associated with neuropathological heterogeneity in aged NCI individuals remains unclear.

To begin to bridge this knowledge gap, we performed total RNA sequencing (RNA-seq) with subsequent bioinformatic inquiry to assess RNA expression level alterations in samples of posterior cingulate cortex (PCC, Brodmann area 23/31) obtained from Rush Religious Orders Study (RROS) participants who came to autopsy with an ante-mortem clinical diagnosis of NCI, but upon post-mortem examination were found to display a range of Braak NFT Stages I–IV (see *Graphical Abstract*).^35^ PCC function plays a role in episodic and autobiographical memory retrieval, attention, salience and emotional memory via unimodal and multimodal inputs from cortical and subcortical modulatory systems as well as prominent efferent projections to hippocampus and other limbic areas.^36-38^ PCC also displays hypometabolic changes,^39-42^ synaptic loss,^43^ and NTF pathology^44^ during the early stages of AD, and individuals who display a higher PCC grey matter volume atrophy rate show a faster decline in multiple cognitive domains.^45^ Moreover, the PCC is a hub of the cortical default mode network (DMN),^46^ which mediates resting state monitoring of the external versus internal environmental milieu.^38,47,48^ Notably, functional MRI studies revealed that functional connectivity of PCC within the DMN is dysregulated during resting states (e.g. internal mentation) and during attention-demanding tasks in MCI and AD,^45,49,50^ whereas the PCC-synchronized degeneration network (SDN),^51^ but not the hippocampus-SDN, associates with AD progression.^49^ Hence, the PCC is a highly suitable region for dissecting molecular changes associated with pathology, cognitive ability and resilience in healthy aged controls.

This RNA-seq study in the PCC revealed 750 genes that were significantly differentially expressed in NCI subjects with Braak Stages III/IV (as a proxy for resilience) compared to those with Braak stages I/II.^35^ Inputting different gene/network data into a functional annotation clustering model revealed elevated pre-synaptic, post-synaptic and ATP-related expression in Braak stages III and IV compared with stages I/II, suggesting these pathways are integral for cognitive resilience seen in unimpaired elderly subjects.^52^ In addition, the resulting analysis revealed eight upregulated and four down-regulated miRNAs in Braak Stage III/IV compared to Stage I/II cases, with miRNA-specific pathway databases predicting functional consequences for axon guidance, long-term potentiation and phosphatidylinositol signalling, among others.^35^

In the present study, we mined this RNA-seq dataset in an unbiased manner to explore associations between specific PCC miRNA transcript levels in each NCI subject and neuropathological burden defined by post-mortem diagnostic criteria, as well as their ante-mortem neuropsychological test scores and demographic variables, including chromosomal sex and age at death. Moreover, we assessed potential interactions between ‘resilience’ associated miRNAs and select mRNA transcripts related to AD-relevant pathways.

## Materials and methods

### Clinical and pathologic assessments of NCI subjects

Post-mortem tissue samples (*n* = 26) were obtained from participants in the RROS, a longitudinal clinical pathologic study of aging and dementia in elderly clergy.^35^ Details of RROS clinical and neuropathologic evaluations and diagnostic criteria are published.^35,53-55^ Briefly, RROS participants undergo an annual neurological examination and cognitive performance testing using the Mini-Mental State Exam (MMSE) and 19 additional neuropsychological tests referable to five cognitive domains: orientation, attention, memory, language and perception.^53^ A composite global cognitive *z*-score (GCS) was derived from this test battery for each subject; NCI subjects did not reveal impairment in any of these domains within a year of death.^53,56^ NCI cases with a history of major depressive disorder, chronic alcoholism and/or neuropathological evidence of Parkinson’s disease, Lewy body disease, TDP-43 proteinopathy, hippocampal sclerosis or large strokes were excluded from the study. Apolipoprotein E (*APOE*) genotyping was performed as reported.^35^

Brain slabs including those containing the PCC were immersion fixed in 4% paraformaldehyde, cryoprotected, cut at 40 µm and sections were immunostained with an antibody against the amyloid precursor protein (APP) and Aβ (6E10, 1:400 dilution) and phosphorylated tau (AT8, 1:250 dilution).^35^ A board-certified neuropathologist evaluated all cases while blinded to clinical diagnosis.^55^ Designations of ‘normal’ (with respect to AD or other dementing processes), ‘possible AD’, ‘probable AD’ or ‘definite AD’ were based on semi-quantitative estimation of neuritic plaque density, an age-related senile plaque score and presence or absence of dementia as established by the Consortium to Establish a Registry for Alzheimer’s Disease (CERAD).^57^ Braak scores based on the staging of NFT pathology were established for each case.^58^ Cases also received a NIA-Reagan Likelihood-of-AD diagnosis based on neuritic plaque and tangle pathology.^59^ The ‘ABC’ algorithm for the diagnosis of AD^60^ is currently being applied to all RROS cases. PCC-specific 6E10 and AT8-immunoreactive burdens were also evaluated using a semi-quantitative score: no 6E10-positive amyloid plaques and no AT8-positive NFTs, neurites or neuropil threads (0) to mild-to-moderate (2–3) to moderate-to-severe (4–5).

### Preparation of tissue, RNA-seq and post-processing

Detailed methodologies for this study have been previously published.^35^ Briefly, PCC samples using fiduciary landmarks defined by the corpus callosum inferiorly, Brodmann area 24 anteriorly and the pre-cuneal cortex ventrally^61^ were flash frozen and stored at −80°C until processing for RNA-seq [Translational Genomics Research Institute (TGen), Phoenix, AZ, USA]. Total RNA was extracted using the mirVana miRNA Isolation Kit (Ambion, Austin, TX, USA) with enrichment for small RNAs, which enabled the assessment of miRNA in addition to mRNA.^62,63^ RNA-seq libraries were prepared using 500 ng of total RNA with the TruSeq Stranded RNA Kit (Illumina, San Diego, CA, USA), ligated with xGen Dual-index UMI adapters (Integrated DNA Technologies, Coralville, IA, USA) and enriched using eight PCR cycles. Libraries were paired end-sequenced on the HiSeq4000 (Illumina) for 80 basepair (bp) reads by TGen.

FastQ files were merged for paired ends prior to quality filtering and trimming using Fast Read Adjustment of Short reads (FLASH-1.2.11, minimum overlap 10 bases, maximum overlap 80 bases, mismatch allowed 1 in 4),^64^ which takes the base with the higher quality where a mismatch occurs. Reads were trimmed (sliding window of 3 bases with an average quality ≥ 32), quality filtered (average trimmed read quality ≥ 30) and size-selected (≥50 bases) using Trimmomatic (0.32).^65^ Reads were mapped to *Homo sapiens* genome Genome Reference Consortium Human Build 38 patch release 13 (GRCh38.p13, hg38, assembly GCF_000001405.39) in Geneious using a custom annotation-span preference algorithm (v.9.0.1; Biomatters, Inc., San Diego, CA, USA) and annotated using feature files for NCBI RefSeq, miRBase, LINC and SNORD/miRNA. Alignment files were exported and raw counts calculated using StringTie (2.1.1).^66^ The experimenter was blinded to all subject data.

### Quantitative real-time PCR

Total RNA was extracted from frozen PCC blocks from the same NCI subjects (*n* = 23/26) using the PureLink RNA Mini Kit (Invitrogen, Carlsbad, CA, USA). MiRNA cDNA synthesis was performed using the TaqMan Advanced miR cDNA Synthesis Kit (Thermo Scientific, Waltham, MA, USA) and mRNA cDNA synthesis was carried out with the iSCRIPT cDNA Synthesis Kit (Bio Rad 1708890), using separate pools of total RNA. Samples were assayed in triplicate using target-specific TaqMan assays for hsa-miR-34a-5p (#478048), hsa-miR-12118 (#483119) or *ADAM10* (#Hs00153853_m1; Applied Biosystems, Foster City, CA, USA) on a QuantStudio 3 real-time PCR instrument (Applied Biosystems). Absolute quantitation was measured by extrapolating Ct values against a standard curve representing a dynamic range of six orders of magnitude.

### Network analysis

Correlations between miRNA expression levels and subject characteristics were performed in an automated Excel spreadsheet that allows for correction of ties, definition of significance correction and output of edge matrix for networks. Formulae were verified in R where applicable with the base stats package (4.1.0 build ‘Camp Pontanezen’). Specifically, cognitive scores were ranked within each test, across subjects, based on performance, with higher rank denoting better performance. In the case of cognitive domains, a composite score from the individual tests was calculated prior to ranking. miRNA post-assembly counts normalized per subject for total miRNA expression level were ranked for miRNA with higher levels of miRNA assigned higher ranks. Ranks were assigned within gene or cognitive test, across all subjects. For example, mir-920 had assigned ranks of 1 to 26, and the cognitive test perceptual speed had ranks of 1 to 26, with one rank for each assigned to a person based on the expression or performance. Ties were corrected using the average rank. Positive correlation denotes an association of increased expression of miRNA with increased performance on tests and negative correlation denotes increased expression of miRNA with decreased performance on cognitive tests. Similarly, higher pathology and increased age were assigned higher ranks, with correction for ties using the mean rank. Spearman rho was used for all network correlations regardless of variable type for the purpose of network modelling. Once correlations were assigned, a cut-off based on uncorrected *P*-values < 0.05 was assigned and miRNA present in <11 subjects were removed. Edge weights were assigned as the absolute value of the correlation multiplied by 100. Gravity network plots were made in Gephi using a two-step gravity loop that applies a separate algorithm at each step with iterations until a limit-cycle or steady-state is reached.^67,68^ Each cognitive domain or pathology score was assigned a node for positive correlations and a separate node for negative correlations and miRNAs were linked accordingly.

### miRNA functional pathway analysis

Functional analysis used a custom reference gene transfer format that included entries from mirBase and RefSeq.^69^ TarBase v8.0 was used as a conservative approach to identify verified, brain-specific miRNA-mRNA interactions with an algorithm-generated prediction score of 0.8.^70^ Pathway analysis of select miRNAs was performed using the Kyoto Encyclopedia of Genes and Genomes (KEGG) via DIANA-mirPath v3.0. Gene Ontology pathways are also available through this resource.^71^ Finally, the StarMir utility^72^ was used to perform a secondary mRNA target prediction analysis for miRNAs that were identified as differentially expressed between low versus high (i.e. ‘resilient’) Braak NFT stages in our previous study.^35^ For this analysis, select genes in specific AD-related pathways were interrogated (see Table 1). Multiple predicted miRNA-mRNA interactions for a single mRNA sequence were counted as a single ‘hit’, and total hits were screened versus a minimum ‘logit probability’ score and adjusted by the number of genes in each category and average to produce an overall ‘Connectivity’ score.

**Table 1:** Genes probed with miRNA sequences

Gene	Accession	Type
ADAM10	NM_001110.4	Amyloid associated
ADAM17	NM_003183.6	Amyloid associated
APP	NM_000484.4	Amyloid associated
BACE1	NM_012104.6	Amyloid associated
CDK5	NM_004935.4	Tau associated
GSK3A	NM_019884.3	Tau associated
GSK3B	NM_002093.4	Tau associated
MAPT	NM_001377265.1	Tau associated
MME	NM_007288.3	Amyloid associated
PSEN1	NM_000021.4	Amyloid associated
PSEN2	NM_000447.3	Amyloid associated
REST	NM_001363453.2	Transcription factor
MAPK13	NM_002754.5	Tau associated
IL1A	NM_000575.5	Cytokine
IL1B	NM_000576.3	Cytokine
IL6	NM_000600.5	Cytokine

### Statistical analysis

Statistical analysis used Spearman rho correlations from network calculations with significance set at 0.05. Numbers are presented as Spearman rho with uncorrected *P*-value. The significance of chromosomal sex correlation with miRNA expression was determined using the point-biserial correlation coefficient in R (ltm 1.2-0) and is presented as a Pearson R with uncorrected *P*-value. Correlations significant at *P* < 0.0005 are highlighted. PCR-based measurements of miRNA or mRNA expression levels were compared among Braak stage groups via Kruskal–Wallis ANOVA with *post hoc* Dunn’'s *z*-test approximation for multiple comparisons. Statistical significance was set at *P* < 0.05.

## Results and discussion

### Subject characteristics

Demographic variables, ante-mortem global cognitive scores (MMSE and GCS) and post-mortem neuropathology diagnostic variables for the 26 NCI subjects are shown in Table 2. As described previously^34^ subjects were divided into three groups: Braak Stages I–II (*n* = 8, 4 M/4F), III (*n* = 8, 3 M/5F) and IV (*n* = 10, 5 M/5F). Although age was significantly different across Braak stages (Kruskal–Wallis *P* < 0.05), no difference was found for MMSE, ApoE genotype, sex, education level, post-mortem interval or CERAD criteria. Significantly greater NIA-Reagan intermediate classifications were found in Braak Stage IV compared to I/II and III (χ^2^, *P* < 0.01, Table 2). PCC amyloid load ranged from absent to moderate in Braak Stages I/II (average score 2.6) and III (2.8) and moderate to severe in Stage IV (4.6; Kruskal–Wallis *P* < 0.01). The average PCC NFT burden ranged from absent to minimal in Stage I/II (average score 0.6) and III (0.7) and minimal to mild in Stage IV (2.2; Kruskal–Wallis *P* < 0.05, Table 2).

**Table 2:** Subject characteristics

	Braak Stage^a^ I–II	Braak Stage III	Braak Stage IV	Chi-square (χ^2^)/Kruskal–Wallis (K)
*n* (male, female)	*n* = 8 (4, 4)	*n* = 8 (3, 5)	*n* = 10 (5, 5)	*P* = 0.84 (χ**^2^**)
Age at death in years (median)	76–92 (79.9)	82–96 (89.1)	83–93 (86.4)	*P* < 0.05 (K)
Education in years (median)	12–21 (15.0)	14–21 (18.5)	14–27 (19.0)	*P* = 0.31 (K)
MMSE score (median)^b^	25–30 (29.0)	26–30 (28.5)	26–30 (28.5)	*P* = 0.89 (K)
GCS^c^ (median)	[−0.32]–[0.42] (0.113)	[−0.14]–[0.43] (0.264)	[−0.55]–[1.55] (0.141)	*P* = 0.70 (K)
ApoE status	ɛ2/ɛ3 *n* = 1	ɛ2/ɛ3 *n* = 0	ɛ2/ɛ3 *n* = 3	*P* = 0.28 (χ**^2^**)
	ɛ3/ɛ3 *n* = 4	ɛ3/ɛ3 *n* = 7	ɛ3/ɛ3 *n* = 5	
	ɛ3/ɛ4 *n* = 3	ɛ3/ɛ4 *n* = 1	ɛ3/ɛ4 *n* = 2	
CERAD^d^	Definite *n* = 1	Definite *n* = 0	Definite *n* = 2	*P* = 0.16 (χ**^2^**)
	Probable *n* = 1	Probable *n* = 2	Probable *n* = 6	
	Possible *n* = 2	Possible *n* = 1	Possible *n* = 0	
	No AD *n* = 4	No AD *n* = 5	No AD *n* = 2	
NIA-Reagan^e^	Intermediate *n* = 1	Intermediate *n* = 2	Intermediate *n* = 8	*P* < 0.01 (χ**^2^**)
	Low *n* = 7	Low *n* = 6	Low *n* = 2	
PCC 6E10 load^f^	2.6 (*n* = 8)	2.8 (*n* = 6)	4.6 (*n* = 10)	*P* < 0.01 (K)
PCC AT8 load^f^	0.6 (*n* = 8)	0.7 (*n* = 6)	2.2 (*n* = 10)	*P* < 0.05 (K)

^a^Braak staging was determined using Bielchowsky silver stain and AT8 immunostaining to identify NFT severity and distribution across the brain. Braak Stages I and II display mild-to-moderate NFTs primarily in the entorhinal cortex; Stages III and IV display a larger involvement into limbic regions including the hippocampus and Stages V and VI revealed moderate-to-severe NFTs across brain regions.

^b^MMSE is a cognitive status exam used to establish a baseline of cognitive function (No dementia = score 26–30).

^c^GCS is derived from 19 cognitive test score including episodic memory, semantic memory, working memory, perceptual orientation and perceptual speed performance.

^d^CERAD based upon post-mortem neuritic plaque pathologic criteria.

^e^NIA-Reagan (National Institute on Aging (NIA) and Ronald and Nancy Reagan Institute of the Alzheimer’s Association (Reagan)) consensus diagnosis of Alzheimer’s disease.

^f^PCC average NFT and plaque load scored from 0-absent to 5-severe. Data were not available for two Stage III cases owing to tissue availability.

### Associations between PCC miRNA levels and subject demographic, cognitive and pathological variables

**Figure 1:**
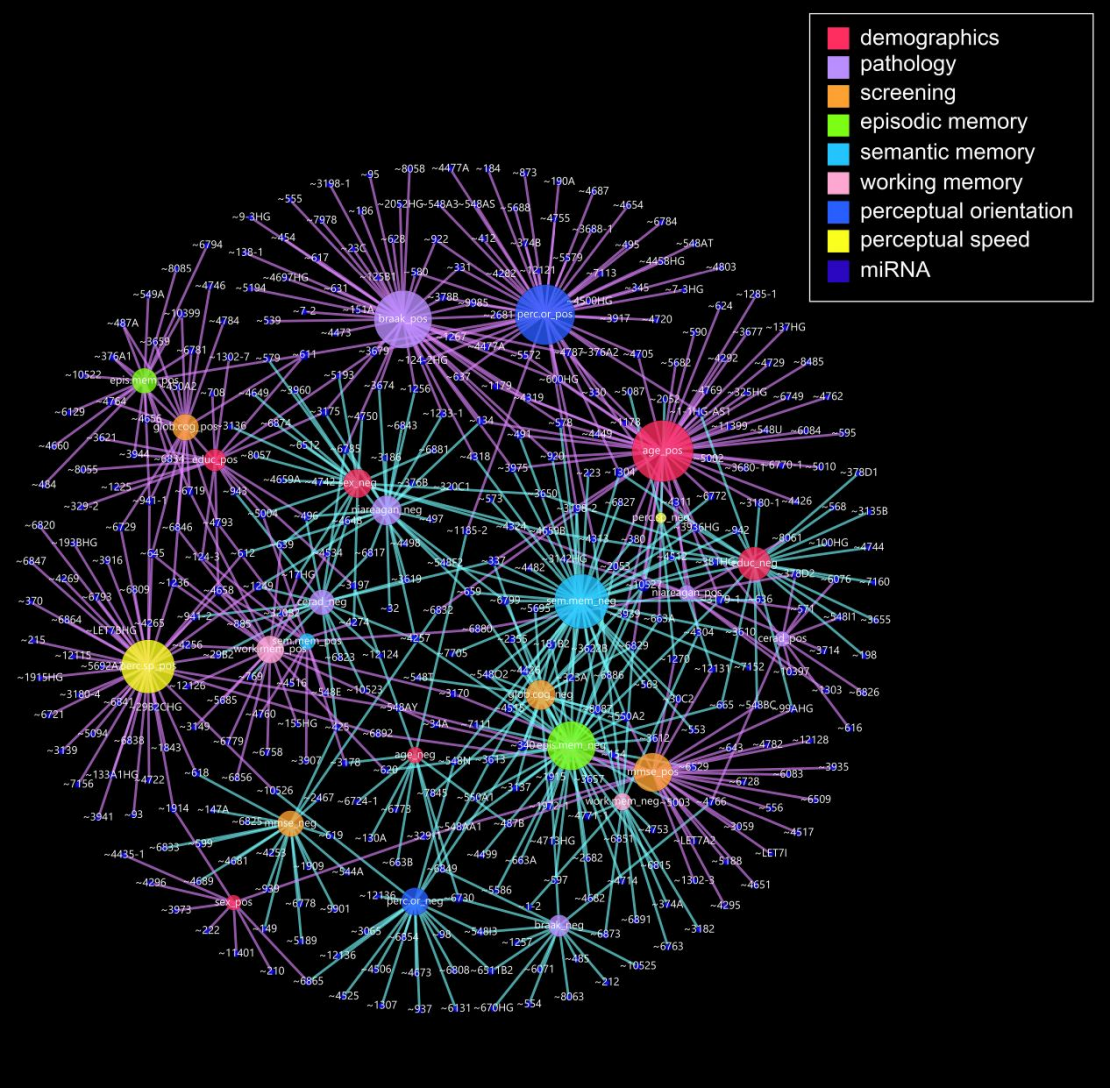
**Network plot of micro-RNA expression level associations with subject data.** A plot was generated using a force-directed algorithm that orders nodes (circles) based on overlap in shared micro-RNA (miRNA). Size of nodes represent number of connections ranging from 5 (perc.sp_neg) to 50 (age_pos). Each edge (line) represents a significant (*P* < 0.05) correlation (Spearman rho) between a miRNA and a hub. Edges are colour-coded to represent positive or negative miRNA expression level correlation with demographics age or education, pathology Braak or NIA-Reagan and patient test scores: screening (MMSE or GCS), episodic memory, semantic memory, working memory, perceptual orientation, or perceptual speed. Details on associations can be found in Supplementary Tables 1 and 2. ∼, miR; _neg, negative correlation with miRNA; _pos, positive correlation with miRNA; age, age at death; braak, Braak score; cerad, Consortium to Establish a Registry for Alzheimer’s Disease pathology score; educ, years of education; epis.mem, episodic memory score; glob.cog, global cognitive score; mmse, Mini-Mental Status Examination; niaregan, National Institute of Aging-Reagan score; perc.or, perceptual orientation score; perc.sp, perceptual speed score; sem.mem, semantic memory score; sex_neg, biological sex male; sex_pos, biological sex female; work.mem, working memory score.

A total of 906 miRNAs were probed for expression levels with an average expression rate of 81% of subjects (range 42–100%). Two hundred and twenty of these were present in all subjects. Secondary analysis of miRNA expression using RNA-seq performed on PCC tissue samples from this cohort,^35^ revealed a total of 667 miRNAs significantly associated (rho > 0.38, *P* < 0.05) with subject demographic, cognitive/neuropsychological and/or neuropathological characteristics (Fig. 1). Notably, there were significant positive correlations between miRNA expression levels and age (50 miRNAs, median rho 0.47, range 0.39–0.72, with three significant at *P* < 0.0005: miR-4762 rho 0.72, miR-578 rho 0.69, miR-4319 rho 0.64), perceptual orientation (49 miRNAs, median rho 0.49, range 0.40–0.69) and perceptual speed (43 miRNAs, median rho 0.48, range 0.39–0.77, with two significant at *P* < 0.0005: miR-1249 rho 0.76, miR-4658 rho 0.66). By contrast, higher miRNA levels correlated negatively with semantic (44 miRNAs, median rho −0.50, range −0.40 to −0.79, with three significant at *P* < 0.0005: miR-3622b rho −0.79, miR-548o2 rho −0.77, miR-10527 rho −0.71) and episodic (39 miRNAs, median rho −0.49, range −0.40 to −0.67) memory (Supplementary Table 1). Moreover, 142 miRNAs significantly correlated with more than one subject characteristic (Supplementary Table 2). These alterations suggest a dynamic restructuring of the transcriptional machinery affecting PCC functionality and different cognitive domains associated with executive function in the face of different levels of NFT pathology in NCI subjects. Specifically, higher expression of miRs-337, 10527 and 6829 was associated with advanced age and poorer performance on more than one cognitive test (e.g. semantic memory); increased expression of miRs-611 and 4658 associated with more years of education and better performance across cognitive domains; and increased expression of miRs-340, 3613 and 8087 were associated with lower performance in three cognitive domains (e.g. episodic and working memory, perceptual orientation) but not with demographic variables (Fig. 1, Supplementary Table 2).

### PCC miRNA expression and performance on specific neuropsychological tests

**Figure 2:**
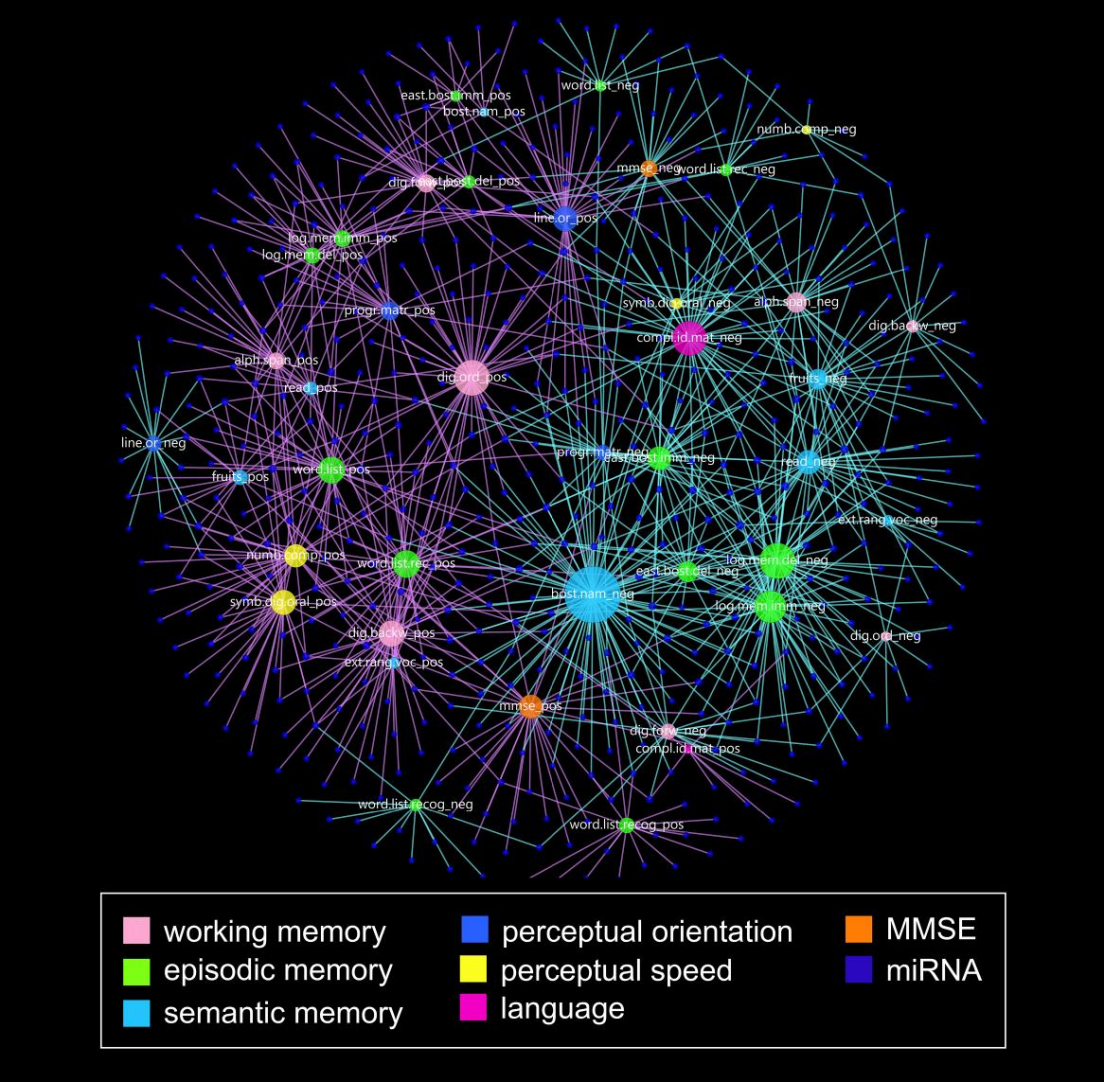
**Network plot of micro-RNA expression level associations with ante-mortem performance on individual neuropsychological tests.** A plot was generated to order nodes (circles) based on overlap to shared micro-RNA (miRNA, smaller blue nodes). Size of nodes represent number of connections, ranging from 5 (perc.sp_neg) to 50 (age_pos). Each edge (line) represents a significant (*P* < 0.05) correlation (Spearman rho) between an miRNA and a hub. Nodes denoting cognitive domains are coloured by cognitive domain and labelled to indicate specific neuropsychological tests within each domain, and edges are coloured to represent positive (purple) or negative (aqua) correlations. Abbreviations: _neg, negative correlation with miRNA; _pos, positive correlation with miRNA; alph.span, alpha span; att, attention; bost.nam, Boston naming 15 items; cat.fluenc, category fluency fruits; chr, chromosome; cog, cognitive test; compl.ideat.matr, complex ideational matrix; dig.forw, digits forward; dig.order, digit ordering; east.bost.del.recall, East Boston delayed recall; epi, episodic memory; ext.rang.voc, extended range vocabulary; lan, language; log.mem.del, logical memory II delayed; log.mem.imm, logical memory I immediate; MIR, micro-RNA; MMSE, Mini-Mental State Exam; numb.comp, number comparison; ori, perceptual orientation; progr.matr, progressive matrices 16 items; progr.matr.subs, progressive matrices subset 9 items; read.test, Reading test 10 items; sem, semantic memory; spe, perceptual speed; symb.dig.oral, symbol digits modality oral; wor, working memory.

Exploring further the relationships between individual miRNA associations with ante-mortem cognitive status, we found significant correlations between miRNAs and specific neuropsychological test scores referable to orientation, attention, memory, language and perception (see Materials and methods) (Fig. 2). A total of 11 miRNAs were identified with expression levels that were either positively or negatively correlated with both MMSE scores individual subscale neuropsychological tests that comprise the GCS (Table 3). For example, miR-34a levels were positively correlated with performance on the MMSE as well as tests associated with semantic memory (extended range vocabulary and reading test 10 items). MiR-6892 levels were also positively correlated with MMSE, but also with perceptual speed and episodic memory. To test whether these associations would remain if PCC miRNA was measured by an independent approach, quantitative real-time PCR (qRT-PCR) was performed in 23/26 cases for miR-34a. We found that significant positive associations were maintained between miR-34a levels and MMSE (*r* = 0.48, *P* = 0.018) as well as performance on reading test 10 items (*r* = 0.42, *P* = 0.041), with a trend for correlation between miR-34a levels and performance on extended range vocabulary (*r* = 0.38, *P* = 0.065). Taken together, these associations are notable due to the lack of cognitive deterioration in this cohort, suggesting that miRNA transcriptional alterations within PCC may influence functional connectivity within hubs of the DMN. We also examined associations between performance on specific neuropsychological tests and subject demographic and biological variables such as age, sex, education level and ApoE genotype (Supplementary Table 3). Except for a positive correlation between higher education level and semantic memory performance, there were no significant differences among the categories. By contrast, multiple significant associations were identified between cognitive test scores and the GCS for each subject.

**Table 3:** miRNA expression correlation with MMSE and neuropsychological tests comprising the GCS in rush religious orders study participants

↑miRNA [chr]	MMSE rho	Cognitive test (rho)	Domain	TarBase 8.0 mRNA interactions for brain
↑MMSE and ↑ cog				
3170 [13]	0.56	↑alph.span (0.75)	wor	
329–1 [14]	0.54	↑cat.fluenc (0.57)	sem/lan	
548ay [3]	0.54	↑numb.comp (0.66)	spe	
6892 [7]	0.52	↑numb.comp (0.40)	spe	
		↑symb.dig.oral (0.43)	spe/att	
		↑word.list.recall (0.43)	epi	
let7a2 [11]	0.51	↑dig.order (0.72)	wor	
34a [1]	0.49	↑ext.rang.voc (0.45)	sem	*ADAM10*, *AP1AR*, *CD47*, *DDX17*, *E2F3*, *FOXJ2*, *GNAQ*, *INA*, *ITGB8*, *MAP2K1*, *MDM4*, *MET*, *MLLT3*, *PDGFRA*, *PEG10*, *PRKCB*, *PRPF38B*, *PTPN4*, *SEPT3*, *SHOC2*
		↑read.test (0.54)	sem	
↓mmse and ↓cog				
599 [8]	−0.58	↓progr.matr (−0.64)	ori	
618 [12]	−0.54	↓compl.ideat.matr (−0.57)	lan	
6833 [6]	−0.51	↓compl.ideat.matr (−0.42)	lan	
6778 [17]	−0.48	↓progr.matr (−0.48)	ori	
		↓progr.matr.subs (−0.43)	ori	
544a [14]	−0.45	↓cat.fluenc (−0.45)	sem/lan	
		↓read.test (−0.50)	sem	

↑ denotes increased performance associates with increased expression of miRNA, ↓ denotes decreased performance associates with increased expression of miRNA.

alph.span, alpha span; cat.fluenc, category fluency fruits; chr, chromosome; cog, cognitive test; compl.ideat.matr, complex ideational matrix; dig.forw, dig.order, digit ordering; epi, episodic memory; ext.rang.voc, extended range vocabulary; lan, language; miRNA, micro-RNA; MMSE, Mini-Mental State Exam; numb.comp, number comparison; ori, perceptual orientation; progr.matr, progressive matrices 16 items; progr.matr.subs, progressive matrices subset 9 items; read.test, reading test 10 items; sem, semantic memory; spe, perceptual speed; symb.dig.oral, symbol digits modality oral; wor, working memory.

### PCC miRNA expression and post-mortem neuropathology in elderly NCI cases

**Figure 3:**
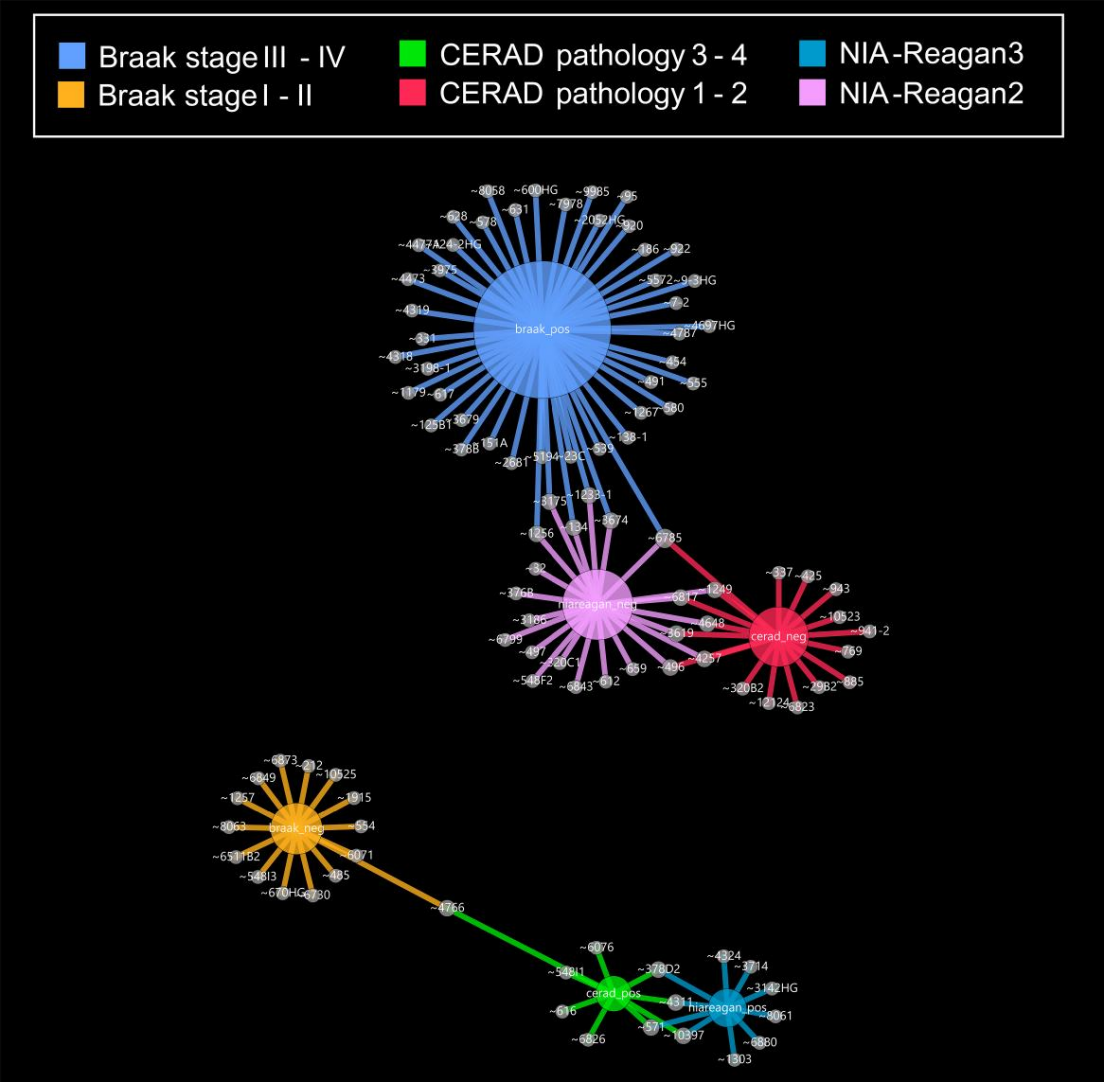
**Network plot of micro-RNA expression level associations with post-mortem neuropathological diagnostic variables.** Plot was generated as noted in Figs 1 and 2 using significant Spearman rho correlations (*P* < 0.05) correlations between higher micro-RNA expression levels and either higher (positive association) or lower (negative association) pathological stage, as indicated (see key). CERAD, Consortium to Establish a Registry for Alzheimer’s Disease; NIA-Reagan, National Institute on Aging-Reagan.

miRNA expression levels correlated with different neuropathological criteria (Fig. 3). Overall, higher expression of 15 miRNAs associated with lower Braak NFT stages (I–II, median rho −0.54, range −0.45 to −0.81, with one significant at *P* < 0.0005: miR-6511b2 rho −0.81) and 47 miRNAs were associated with higher Braak stages (III–IV, median rho 0.46, range 0.39–0.88 with one significant at *P* < 0.0005: miR-331 rho 0.88). Indeed, there was a notable enrichment of miRNAs with expression levels that correlated positively with Braak Stages III–IV (Fig. 3), suggesting these associations may be related to resilience in the face of mounting AD neuropathology.^35^ By contrast, 18 miRNAs associated with lower CERAD neuritic plaque burden (no/possible AD, median rho −0.49, range −0.41 to −0.70) compared to nine miRNAs associated with higher CERAD neuritic plaque burden (probable/definite AD, median rho 0.50, range 0.45–0.81). Considering NIA-Reagan criteria, which includes NFT and plaque burden, 10 miRNAs were associated with ‘low likelihood of AD’ (Supplementary Table 2). Interestingly, when multiple neuropathological measures were associated with a particular miRNA, CERAD criteria were discordant with Braak or NIA-Reagan scores (Supplementary Table 2, Fig. 3).

### miRNA gene target and bioinformatic pathway analysis

TarBase 8.0, which is curated based on validated miRNA-mRNA interactions reported in the literature,^72^ was selected as a conservative but rigorous approach for target analysis. We first searched for brain-specific interactions (e.g. *in vitro* regulation in primary cortical neurons) for miRNAs that positively correlated with MMSE scores as well as with other neuropsychological tests (Table 3). Interestingly, only mRNAs validated as targets of miR-34a-5p were identified (Table 3). miR-34a plays a known role in tumour suppression^73^ but is increasingly associated with vascular aging and cell senescence^74^ as well as a suppressor of neuronal apoptosis.^75^ Pathway analysis based on predicted gene interactions of the miRNAs in this subset included many related to prion disease, fatty acid biosynthesis and metabolism, hippocampal signalling pathways, cancer progression including cell cycle regulation, and lysine degradation, which is an important upstream step in mitochondrial respiration (Supplementary Table 4).^76^ The identification of prion disease as a top functional pathway was intriguing given the prion-like properties of misfolded tau proteins^77^ and suggest that miRNAs may be involved in regulating the pathogenic pathways leading to aberrant tau proteoforms (e.g. trafficking and degradation),^78^ which may influence PCC functional activity during cognitive processes. Moreover, a recent study of age-related alterations in plasma miRNAs in a normative aging cohort of men found that gene targets of miRNA that correlated with changes in MMSE scores over time were associated with prion disease, as well as with fatty acid biosynthesis pathways.^79^

We next queried for mRNA targets of miRNAs that were negatively correlated with MMSE scores and positively or negatively related with other neuropsychological variables (Table 3), using the same algorithm filters in TarBase as described above. No mRNA targets were identified, but KEGG pathway analysis showed that miRNAs within this subset were predicted to regulate functions such as lysine degradation, fatty acid biosynthesis and metabolism, hippocampal signalling and cell cycle activity (Supplementary Table 5). Finally, we searched for target mRNAs for all miRNAs associated with neuropathological criteria (Supplementary Table 2). No mRNA targets were identified, yet KEGG analysis predicted functionality for this miRNA subset in processes related to lysine degradation, fatty acid biosynthesis and metabolism, as well as p53 signalling (Supplementary Table 6). Hence, unbiased bioinformatic inquiry revealed pathways related to lysine degradation/respiration and fatty acid cell biology as two common functional threads among the three miRNA clusters.

### Associations between resilience-related PCC miRNAs and AD-related genes

**Figure 4:**
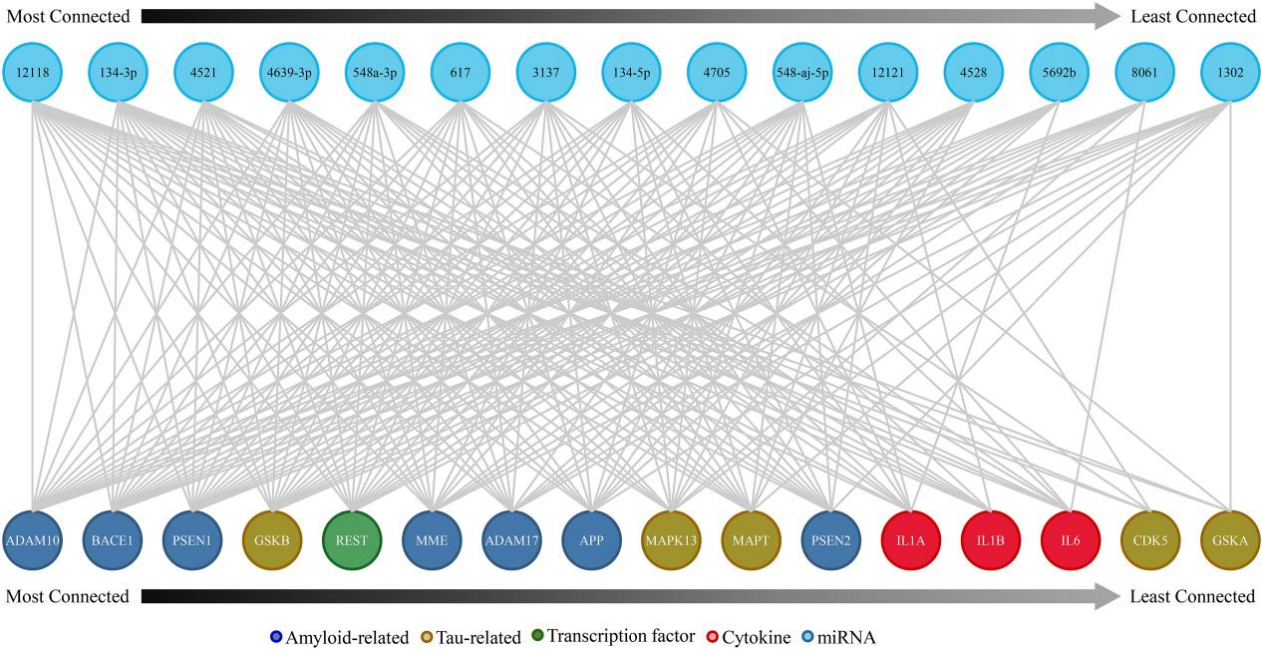
**Co-regulatory network predicted for resilience-related PCC micro-RNAs and AD-related genes.** Micro-RNA (miRNA) sequences related to resiliency in PCC were used to probe the StarMir database for potential mRNA targets known to have influence on AD pathogenesis and progression. Sixteen mRNA targets related to amyloidosis (ADAM19, BACE1, PSEN1, MME, ADAM17, APP, PSEN2), tau and its aggregation (GSKB, MAPK13, MAPT, CDK5, GSKA), a transcription factor (REST) and cytokines (IL1A, IL1B, IL6) appeared in the search, each potentially regulated by 4–15 miRNA species.

**Figure 5:**
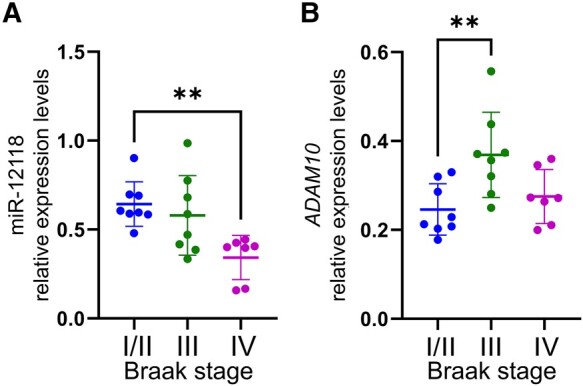
**qRT-PCR validation of select micro-RNAs and predicted mRNA targets** (**A**) miR-12118 and (**B**) *ADAM10* transcript expression levels measured in PCC samples from NCI cases, subdivided into Braak Stages II/II, Braak Stage III or Braak Stage IV. **, *P* < 0.01 via Kruskal–Wallis ANOVA with *post hoc* Dunn’s test for multiple comparisons.

A total of eight miRNAs were significantly upregulated (miR-12121, miR-134, miR-3137, miR-4528, miR-4639–3p/548a-3p cluster, miR-4705, miR-5692b and miR-617) and four miRNAs were significantly down-regulated (miR-12118, miR-1320/8061 cluster, miR-4521 and miR-548aj-5p) in PCC when comparing Braak Stage III/IV or IV to Braak Stage I/II cases.^35^ To examine whether these miRNAs were related to protein dysregulation during the progression of AD, we used the StarMir prediction algorithm to probe for specific miRNA binding sites on transcripts encoding established AD-related proteins (Table 1) and the relative probability that multiple predicted miRNA-mRNA interactions occur for a single mRNA sequence, which resulted in network ‘connectivity’ scores. AD-related mRNAs with multiple, high probability binding sequences for the dysregulated miRNAs included those related to amyloid processing (e.g. *BACE1* and *ADAM10*), tau (e.g. *GSK3B* and *CDK5*), cytokines (e.g. *IL1B* and *IL6*) and the *REST* transcription factor (Fig. 4). Among its multiple statistics, StarMir reports a logit probability that a prediction will be valid (Supplementary Table 7). qRT-PCR validation studies showed that levels of miR-12118 were down-regulated by ∼35% in Braak Stage IV compared to Braak Stage I/II cases (Fig. 5A), similar to the ∼20% downregulation previously measured between Braak Stage IV compared to Braak Stage I/II cases by sequencing.^35^ Additionally, we noted that *ADAM10*, which codes for the metalloproteinase ADAM10, was highly connected within the resilience-related miRNA network. ADAM10 has been identified as the primary α-secretase of APP resulting in soluble, non-amyloidogenic APP processing,^80,81^ which plays a role in regulating amyloid pathology. Furthermore, conditional knockout of *Adam10* in mice induces altered post-synaptic spine morphology and a reduction in cell-surface NMDA receptors, with concurrent memory deficits and seizures.^82^ To examine *ADAM10* mRNA expression in the NCI cases, we performed qRT-PCR and found that *ADAM10* was significantly upregulated by ∼50% in Braak III cases compared to Braak I/II cases, with these levels returning close to baseline in Braak Stage IV (Fig. 5B). This transient increase may represent an innate compensatory or plasticity response in the PCC in the face of mounting AD pathology in PCC in an attempt to maintain cognitive performance.

In summary, we demonstrate miRNA levels associated with age, education level, cognitive performance and post-mortem neuropathological AD diagnostic criteria in the PCC hub of the DMN memory circuit in cognitively intact elders. Significantly, these subjects passed exclusion criteria ruling out possible pathology associated with non-AD dementia, suggesting the relevance of these miRNA associations for gene regulatory mosaics associated with aging, pre-clinical AD and possibly even resilience to the onset of AD pathology. In this regard, the predicted regulatory targets of miRNAs correlating with either neuropsychological test scores or AD neuropathological criteria belonged to functional gene classes operating within pathways mediating lysine degradation, fatty acid biosynthesis and metabolism.

The main pathway for lysine degradation begins with ɛ-deamination of the amino acid via the mitochondrial saccharopine pathway, which consists of nine enzymatic steps that ultimately yield two acetyl-CoA units and several reducing equivalents critical for the citric acid cycle and redox homeostasis.^76^ Intriguingly, among several functional roles for free lysine in cell biological processes, including those related to peroxisome function,^83^ the acetyl-CoA products from lysine degradation also provide the essential substrates for fatty acid biosynthesis.^84^ With respect to lipid metabolism, the link between demographic, genetic and lifestyle factors affecting lipid biosynthesis and degradation disruption and AD risk are well-established, including allelic inheritance of APOE isoforms modulating lipid transport and turnover.^85,86^ Dysregulation of free fatty acid and phospholipid levels as well as lipid peroxidation have been observed in brain and plasma in MCI and AD.^87-89^ Mechanistically, these phenomena have been linked to mitochondrial uncoupling, which lowers cellular energy production,^84,90^ free arachidonic acid- and phospholipase 2A-mediated pro-inflammation^91^ and a disruption of lipid raft efficiency to facilitate synaptic transmission.^92,93^ With respect to AD pathology, increasing cholesterol synthesis by overexpressing SREBF2 in APP/PS1 transgenic mice accelerated amyloid pathology and cognitive decline, but also induced endogenous tau hyperphosphorylation and NFT-like inclusions.^94^ By contrast, lower polyunsaturated fatty acid levels were correlated with lower Aβ_1–42_/total tau levels in cerebrospinal fluid of NCI subjects suggesting a putative role for lipid metabolism in resilience.^95^ Taken together, these data suggest that differential expression levels of select miRNAs in the PCC play a key role in the regulation of metabolic functions that affect cognitive performance and AD pathological processes in elderly NCI subjects. Additional evidence indicates that resilience-related miRNAs also target mRNAs coding for APP and tau and their metabolic proteins as well as AD-relevant inflammatory cytokines. Hence, this study highlights the potential pathogenic role of miRNAs—and other non-coding RNAs—in cognitive resilience and during the pre-clinical/prodromal stages of AD.^25-34,96,97^ Along these lines, circulating miRNAs may serve as reliable biomarkers for cognitive decline in AD and related dementias.^98,99^ Pre-clinical studies showed that *in vivo* overexpression of miR-31 lowered APP and BACE expression to reduce amyloid pathology and improve cognition in 3xTg-AD mice.^100^ By contrast, inhibiting miR-146a with a selective antagomir increased ROCK1 levels, reduced tau hyperphosphorylation and improved cognition in 5XFAD mice.^101^ Currently, non-surgical miRNA-based therapies are not being tested clinically,^102^ but strategies such as nasal delivery of nanoparticle miRNAs are under development in the cancer therapeutics space.^103^ To date, nasal administration of a long non-coding RNA was reported to regulate insulin signalling and reduce inflammation in aged mice in the absence of toxicity or adverse events.^104^ Finally, an exciting new line of investigation involves identifying non-coding RNAs that regulate miRNA expression and activity, such as circular RNAs.^105^

Study limitations include the sample size, although the number of significant subject-trait associations detected within the false discovery rate boundaries of FDR = 0.1 was compelling given the statistical stringency of the analysis. Furthermore, the very nature and evolutionary origins of miRNA present drawbacks for building consensus annotation files: genetic clusters can contain multiple overlapping miRNAs co-expressed with long non-coding RNA (e.g. MIR99AHG),^106^ and repetition of genetic elements within gene clusters and across chromosomes can result in splitting counts across multiple regions. In the present study, we used a composite annotation file and a mapping method that iterates and resolves ambiguities using a random seed to correspond more broadly with prior literature and work conducted in other labs, which came at the cost of a finer resolution in a small cohort. We provide novel information indicating pathways relevant to potential early protective (resilience) or pathogenic (pre-clinical) roles of select miRNA-mRNA interactions supporting our previous RNA-seq expression data in the PCC related to brain resilience in cognitively intact elders.^35^ Future research would benefit from an analysis of other cortical DMN (e.g. pre-cuneus and frontal cortex) and limbic (e.g. hippocampus) regions, which also displays a range of NFT pathology in non-demented elderly people. In addition, an analysis of archived gene expression datasets, as well as functional validation in mixed human brain cultures and animal models,^27,33^ are needed to gauge the potential for targeting these regulatory interactions as protective therapies against cognitive decline very early in the onset of AD.

## Supplementary Material

fcae082_Supplementary_Data

## Data Availability

Data are available upon request to the corresponding author and stored in the GEO Database (data submitted to GEO for accession number, which will be provided for publication). Files document multiple steps in the process to aid further research and cross-validation efforts.
